# Study of Building Safety Monitoring by Using Cost-Effective MEMS Accelerometers for Rapid After-Earthquake Assessment with Missing Data

**DOI:** 10.3390/s21217327

**Published:** 2021-11-03

**Authors:** Jian-Fu Lin, Xue-Yan Li, Junfang Wang, Li-Xin Wang, Xing-Xing Hu, Jun-Xiang Liu

**Affiliations:** 1 MOE Key Laboratory for Resilient Infrastructures of Coastal Cities, College of Civil and Transportation Engineering, Shenzhen University, Shenzhen 518060, China; 2 Center of Safety Monitoring of Engineering Structures, Shenzhen Academy of Disaster Prevention and Reduction, China Earthquake Administration, Shenzhen 518003, China; linjf@szadpr.cn (J.-F.L.); wlx@szadpr.cn (L.-X.W.); ljx@szadpr.cn (J.-X.L.); 3 MOE Key Laboratory of Disaster Forecast and Control in Engineering, School of Mechanics and Building Engineering, Jinan University, Guangzhou 510632, China; celixy@jnu.edu.cn; 4 Institute of Geophysics, China Earthquake Administration, Beijing 100081, China; huxx@cea-igp.ac.cn

**Keywords:** cost-effective MEMS accelerometer, building safety monitoring, missing data reconstruction, structural health monitoring, after-earthquake assessment

## Abstract

Suffering from structural deterioration and natural disasters, the resilience of civil structures in the face of extreme loadings inevitably drops, which may lead to catastrophic structural failure and presents great threats to public safety. Earthquake-induced extreme loading is one of the major reasons behind the structural failure of buildings. However, many buildings in earthquake-prone areas of China lack safety monitoring, and prevalent structural health monitoring systems are generally very expensive and complicated for extensive applications. To facilitate cost-effective building-safety monitoring, this study investigates a method using cost-effective MEMS accelerometers for buildings’ rapid after-earthquake assessment. First, a parameter analysis of a cost-effective MEMS sensor is conducted to confirm its suitability for building-safety monitoring. Second, different from the existing investigations that tend to use a simplified building model or small-scaled frame structure excited by strong motions in laboratories, this study selects an in-service public building located in a typical earthquake-prone area after an analysis of earthquake risk in China. The building is instrumented with the selected cost-effective MEMS accelerometers, characterized by a low noise level and the capability to capture low-frequency small-amplitude dynamic responses. Furthermore, a rapid after-earthquake assessment scheme is proposed, which systematically includes fast missing data reconstruction, displacement response estimation based on an acceleration response integral, and safety assessment based on the maximum displacement and maximum inter-story drift ratio. Finally, the proposed method is successfully applied to a building-safety assessment by using earthquake-induced building responses suffering from missing data. This study is conducive to the extensive engineering application of MEMS-based cost-effective building monitoring and rapid after-earthquake assessment.

## 1. Introduction

**Figure 1:**
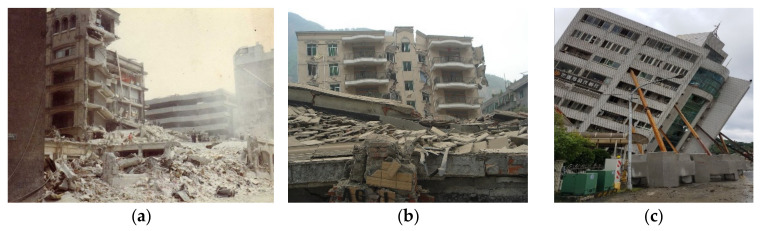
Typical damages of building after earthquakes. (**a**) Damaged building in 1985 Mexico city earthquake. (**b**) Damaged building in 2008 Beichuan earthquake. (**c**) Damaged building in 2018 Hualien earthquake.

Civil structures, such as buildings, bridges, and tunnels working in the natural environment, suffer from structural deterioration and even natural disasters (e.g., earthquakes and typhoons) in their long-term service life. As a result, the resilience of civil structures to extreme loadings drops inadvertently, which may lead to catastrophic structural failure and present great threats to public safety. Earthquake-induced extreme loading is one of the major reasons for the structural failure of buildings, and some typical damages to buildings due to seismic disasters are shown in Figure 1. To reduce the seismic risk to buildings, three scales of monitoring approaches, namely country-, urban-, and building-scale monitoring networks, are established for different tasks [1,2]. Country-scale seismic monitoring networks have been well-established in many earthquake-prone countries, including the USA, China, Japan, and Italy. These national networks are intended to determine the occurrence time, epicenter location, and magnitude of an earthquake. Later, urban-scale networks are developed for mapping higher-spatial-resolution earthquake intensity and implementing early earthquake warning (EEW) systems. Recently, building-scale networks are further conceived for structural health monitoring (SHM) and damage assessment with different assessment indexes [3,4,5]. The three scales of the seismic monitoring network are characterized by different coverage areas and densities of sensor nodes. It is noted that the sensor density in a building-scale network is normally much higher than that of an urban- or country-scale network. Therefore, the application of building-scale monitoring is limited, due to the need for a great number of sensors and the associated high price of SHM systems.

The low price of Micro-Electro-Mechanical System (MEMS) sensors makes it affordable for general-purpose applications and can potentially lead to the popularization of dense seismic monitoring. Over the past ten years, cost-effective MEMS accelerometers have been investigated for recording strong seismic motion and conducting dense EEW. American researchers [6,7,8] proposed a novel MEMS-based strong-motion seismic network named the Quake-Catcher Network (QCN) for community participation in earthquake monitoring, targeted at providing an advance alert for an associated seismic hazard. Moreover, other studies explored the possibility of using the MEMS accelerometers integrated within smartphones to develop citizen-engaging networks for earthquake observation and EEW [9,10]. Nof and Chung et al. [11] proposed an array-based approach to improve early-warning performance using mini-array low-cost MEMS accelerometers. Following the establishment of a high-density seismic network equipped with a low-cost MEMS system called the P-wave-alert-device (P-Alert) by the earthquake early-warning research group at National Taiwan University [12,13], scholars from Taiwan investigated its applicability to dense EEW [12,13,14]. Among them, Jyh and Wu et al. [14] demonstrated that the P-Alert system can determine the near-real-time coseismic deformation (Cd) values as accurately as using GPS and TSMIP stations. Later, Fu and Meng et al. [15] evaluated the performance of low-cost MEMS seismic sensors for dense EEW, based on the 2018–2019 field testing data in Southwest China. The tests have shown that the low-cost MEMS seismic sensors can obtain clear seismic phases and thus trigger earthquake detections for EEW. Pierleoni and Marzorati et al. [16] proposed a low-cost MEMS accelerometric unit and have conducted field tests in Italy to compare the performance of a seismic station using the MEMS accelerometric unit and that of an INGV high-performance station. It was found from the comparative analysis that the proposed MEMS sensors exhibited performances very close to those of more sophisticated and expensive devices and could satisfactorily meet the requirements of seismic monitoring and early warning. The above applications validate the effectiveness of MEMS sensors for urban-scale seismic monitoring, which aims at higher-spatial-resolution seismic intensity identification and the potential for indirect health condition estimation of buildings in the monitored urban area. The indirect health condition estimation is a rough estimation approach by only measuring ground motion to assess the seismic intensity and then indirectly predicting the potential health state of buildings in the monitored urban area. The prediction also depends on statistical health-condition data of different kinds of buildings under different seismic intensities.

Different from the EEW systems that installed MEMS sensors on the ground, the building-scale seismic monitoring systems are characterized by the distributive deployment of MEMS sensors on multiple floors of a building. Due to the high costs of the monitoring systems and their installation, management, and maintenance, building owners are generally reluctant to install permanent monitoring systems, and most seismic damage assessment of the building is currently conducted by technicians’ visual inspection. To overcome this limitation, extensive efforts have been devoted to pre-studies on the applicability of cost-effective MSMS sensors to earthquake-induced building damage assessment [17,18,19,20]. Liang et al. developed a MEMS-based SHM system and shaking table tests were conducted on a small-scale three-story specimen [21]. By comparing cost-effective MEMS accelerometers with the traditional force-balance principle-based accelerometers characterized by high cost and accuracy, the experimental studies showed that MEMS accelerometers were applicable for accurate acceleration response measurement and structural frequency computation under relatively strong vibration. To guarantee the safety and serviceability of buildings in Italy, the EU-funded MEMSCON project aims to produce small-sized MEMS sensors for the measurement of strain and acceleration. In the MEMSCON project, Pozzi and Zonta et al. [22] conducted comprehensive laboratory tests and proved both the developed strain gauge and the accelerometer networks were reliable under operational conditions close to those in a field application. Ha et al. [23] developed a MEMS inclinometer for structure health monitoring and conducted an experimental validation study on a beam model. This type of sensor has the potential to measure the earthquake-induced absolute inclination of buildings. Yin and Wu et al. [24] conducted a pre-study to examine the SHM performance of MEMS sensors. It validated that the low-cost MEMS-type seismometer is reliable to measure the responses of an eight-story one-quarter-scale steel frame structure and the fundamental normal-mode frequencies can be successfully estimated from the measured responses. Fu et al. [25] proposed a demand-based wireless MEMS sensor to meet the requirements of sudden event monitoring with a minimal budget, and the experimental results have shown that the proposed system was able to capture the occurrence of sudden events and provide high-fidelity data for structural condition assessment. Ting and Ren et al. [26] conducted a series of steel-frame shaking table tests with incremental damage and confirmed that the P-alert system was effective for evaluating post-earthquake building safety.

The aforementioned investigations for MEMS-based building-scale seismic monitoring mainly focus on sensor development and laboratory tests for sensor performance validation and/or structural health assessment. To bridge the gap between laboratory exploration and engineering applications, some researchers focus on the on-site application of MEMS-based cost-effective SHM systems. Losanno and Londono et al. [27] presented a monitoring system for a worship building and the system was included in the network of the Italian Observatory of Structures. This investigation demonstrated that the system was able to timely monitor the structural response under extreme events and give rapid assessment results for emergency management. Potenza et al. [28] conducted long-term structural monitoring of a church with a low-cost wireless MEMS sensor network, and the modal parameters were successfully extracted for damage assessment. 

To proceed with structural damage detection of civil structures, many vibration-based damage detection methods have been developed [29,30,31,32] using a limited number of sensors. The basic idea behind these investigations is that changes in the physical properties are associated with changes in the modal properties and the vibration responses. A sensitivity-based model-updating method with different damage detection indexes are usually used for detecting, localizing, and quantifying defects in structures [33,34,35,36,37,38]. However, the sensitivity-based model-updating methods often rely on the input of artificial excitation and a finite element model of the monitored civil structure. This requirement of a large amount of artificial energy input is difficult to realize for a large civil engineering structure, and the establishment of its finite element model is also very time-consuming. To overcome the requirement of artificial excitation, Law et al. [39,40,41] proposed a structural condition assessment approach based on ambient excitation, which relies on the measured acceleration responses of the structure before and after damage occurs to identify structural damage. More recently, Lin and Xu et al. [42,43,44] proposed the use of multiple types of sensors and a multi-scale finite element model for more accurate damage detection of large civil structures. Nonetheless, these sensitivity-based model-updating methods still need to build a complicated finite element model for the civil structures and the updating progress is usually very time-consuming. Different from previously mentioned structural damage-detection methods relying on offline calculation or finite element models, the first-order eigen-perturbation techniques have been developed for real-time damage detection of vibration systems and a comprehensive review can be found in Ref. [45]. To foster the development of real-time eigen perturbation methods, Bhowmik and Krishnan et al. [46,47,48,49,50] successively proposed multiple advanced algorithms incorporating the recursive principal component analysis (RPCA), RPCA-time-varying auto regressive (TVAR), recursive singular spectrum analysis (RSSA), and recursive canonical correlation analysis (RCCA). They are novel baseline-free approaches for continuous online damage detection, able to decrease computational complexities and resource consumption compared with adaptive filtering methods such as the traditional Kalman filter. To demonstrate the effectiveness of the abovementioned real-time eigen perturbation methods for structural health assessment, numerical and experimental case studies were undertaken for the damage detection of single-degree-of-freedom oscillators and multiple-degrees-of-freedom beam models. The case studies indicated that these real-time eigen perturbation methods can accurately identify the damage occurrence of the oscillators and the beam models by using one accelerometer or limited numbers of accelerometers.

To sum up, SHM is a cutting-edge solution for structural safety assessment, but it often relies on expensive sensory systems cooperating with complicated damage detection methods and finite element models. The prevalent SHM systems are generally very expensive and complicated for extensive application, and thus many buildings in earthquake-prone areas of China lack safety monitoring. Moreover, most investigations in the SHM field are mainly confined to laboratory exploration, in which simplified building models or small-scaled frame structures subjected to artificial excitations are employed for experimental studies. With such a large difference from the fundamental frequencies of real buildings and their real operational conditions, the results of these vibration-based structural health monitoring investigations may not be extendable to in-service buildings. In order to promote the extensive application of SHM technology to in-service buildings in earthquake-prone areas, this paper proposes a building-safety monitoring and assessment scheme using cost-effective MEMS accelerometers and examines its effectiveness with the use of an in-service building subjected to real earthquakes.

The main contribution of this paper is the proposal and application study of a rapid after-earthquake assessment scheme for building-safety assessment using cost-effective MEMS accelerometers, which aims to facilitate rapid after-earthquake building assessment for engineers. Specifically, the contribution of this study lies in multiple aspects, including (i) offering parameter analysis of the cost-effective MEMS sensors and suitability assessment of the sensors for building-safety monitoring; (ii) conducting an analysis of seismic risk in China, and providing a distribution map of China’s active seismic faults, a historical seismic event distribution map in China during 2009–2020, and an analysis of the number and proportion of the historical earthquakes with five different magnitudes in China during 2009–2020, which reveal the necessity of developing a cost-effective monitoring system and a rapid building-safety assessment scheme and the necessity of conducting an application study using a real building; (iii) proposing a building safety assessment scheme that systematically consists of fast missing data reconstruction, fast displacement estimation based on the frequency-domain integral of acceleration response, and maximum displacement and maximum inter-story drift ratio (IDR) based assessment; and (iv) instead of performing experimental studies in the lab using scaled models and artificial excitations, this paper carries out an application study using a typical in-service building selected from an earthquake-prone area based on an analysis of seismic risk in China for validating the proposed rapid after-earthquake assessment scheme in a missing data situation.

The rest of this paper is organized as follows. Section 2 describes the establishment of the MEMS sensor-based building monitoring system. Section 3 presents the methodology for rapid after-earthquake assessment using data from the MEMS sensor-based cost-effective structural health monitoring system. Section 4 provides a case study to validate the assessment scheme including fast missing data reconstruction, fast displacement estimation from the acceleration response, and the maximum displacement and maximum IDR-based assessment. It is followed by Section 5 with further discussion. The conclusions are drawn in Section 6.

## 2. MEMS Sensor-Based Building Monitoring System Establishment

### 2.1. Parameter Analysis of a Cost-Effective MEMS Sensor

**Figure 2:**
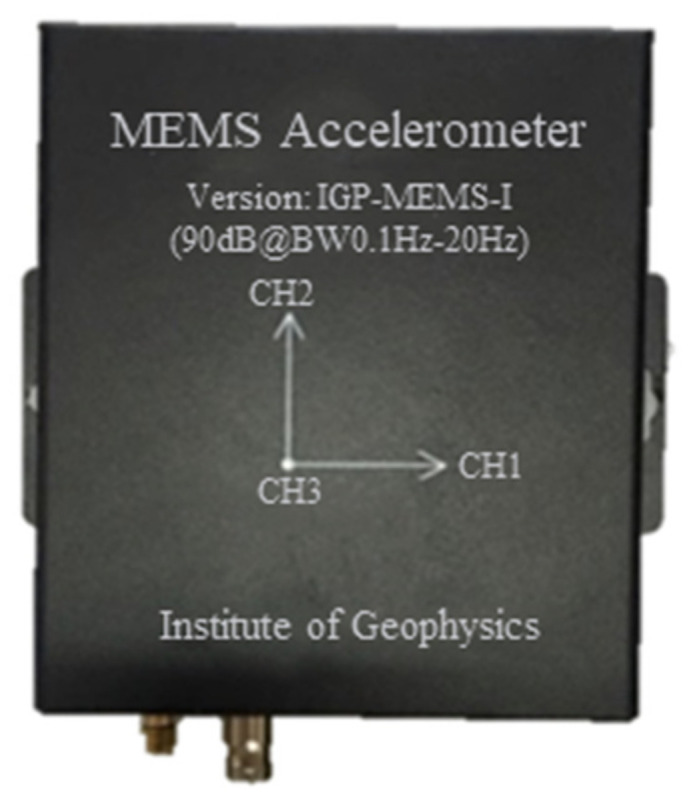
The triaxial cost-effective MEMS accelerometer.

A typical MEMS sensor can integrate the sensing module, filter, A/D conversion module, control module, and data logger in a single circuit board, thus these types of sensors can lead to a cost-effective application to building safety monitoring. However, the accurate measurement and signal processing of small-amplitude low-frequency vibration from civil structures are considerably more challenging than the measurement of strong or high-frequency vibration information from mechanical engineering [51], which makes many MEMS sensors developed by mechanical engineers inapplicable to SHM of civil structures. To collect earthquake-induced vibration and realize rapid after-earthquake assessment of a building, the cost-effective MEMS sensors (see Figure 2) developed by the Institute of Geophysics affiliated with the China Earthquake Administration are examined in this study.

**Figure 3:**
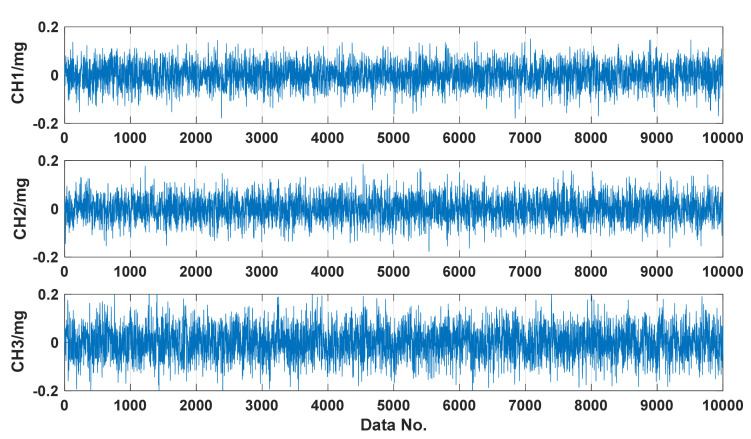
A typical record of measurement noise for the MEMS sensor.

**Figure 4:**
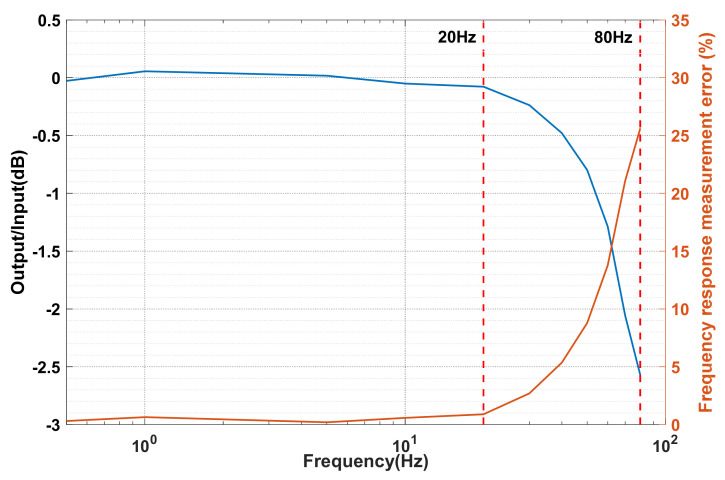
Typical curves of frequency response and the corresponding measurement error for the MEMS sensor.

**Figure 5:**
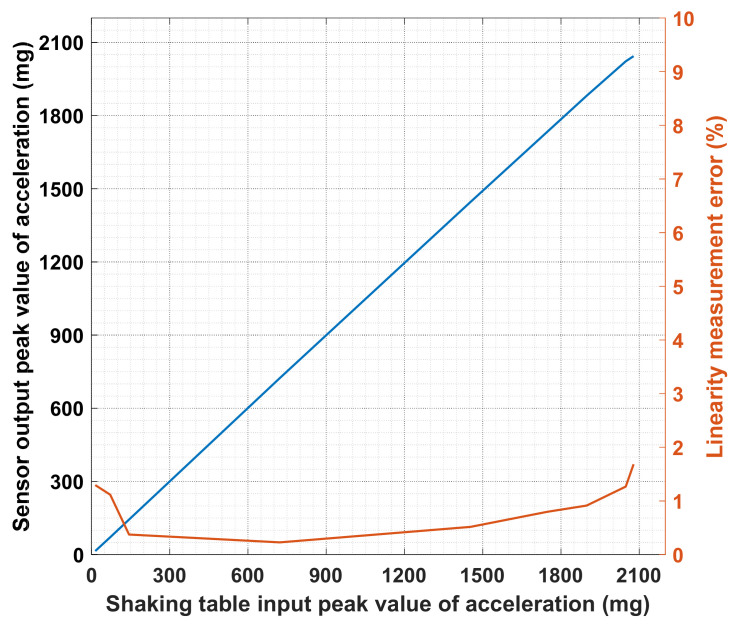
Typical curves of the linearity and the corresponding measurement error for the MEMS sensor.

Parameter analysis of the cost-effective MEMS sensors should be firstly conducted to confirm their suitability for building-safety monitoring. To examine the typical properties of the MEMS accelerometers, the noise level, frequency response, and linearity are tested by installing the sensor on an observation pier connected to bedrock in a laboratory. Figure 3 shows a typical record of measurement noise acquired in the sensor test. The signal-to-noise ratio is computed as 92 dB according to the equation SNR = 20 × lg (2000/√2/0.035) ≈ 92 dB, where 2000 mg is the measurement range and 0.035 mg is the effective value of the measured noise. Moreover, a frequency response test and a linearity test are conducted, and the results are depicted in Figure 4 and Figure 5. Typical curves of the frequency response and the corresponding measurement error for the MEMS sensor are shown in Figure 4. It is found from the frequency response (blue curve) that the −3 dB frequency band is up to 80 Hz. Since civil structures’ low-order vibration modes are dominant and the high-order vibration modes are difficult to excite, a measurement bandwidth up to 20 Hz can meet the measurement requirement of civil structures. The frequency response measurement error (orange curve) is defined as the absolute value of the relative difference between the input and output response. The typical measurement error of frequency response in the frequency band up to 20 Hz is lower than 1% as shown in Figure 4. Similarly, typical curves of the linearity and the corresponding measurement error for the MEMS sensor are shown in Figure 5. The high linearity between the input and output can be observed and the measurement range can reach ± 2000 mg. The linearity measurement error (orange curve) is defined as the absolute value of the relative difference between the input and output response, and it is observed that the typical measurement error of linearity is lower than 2% as shown in Figure 5. As proven, the selected MEMS sensors have a low noise level and low measurement error in the frequency band of interest, and thus are suitable for the seismic response measurement of the building. The typical values of the above core technical parameters and other parameters of the MEMS sensor are summarized in Table 1.

### 2.2. Analysis of Seismic Risk in China and the Selected Building for Case Study

**Figure 6:**
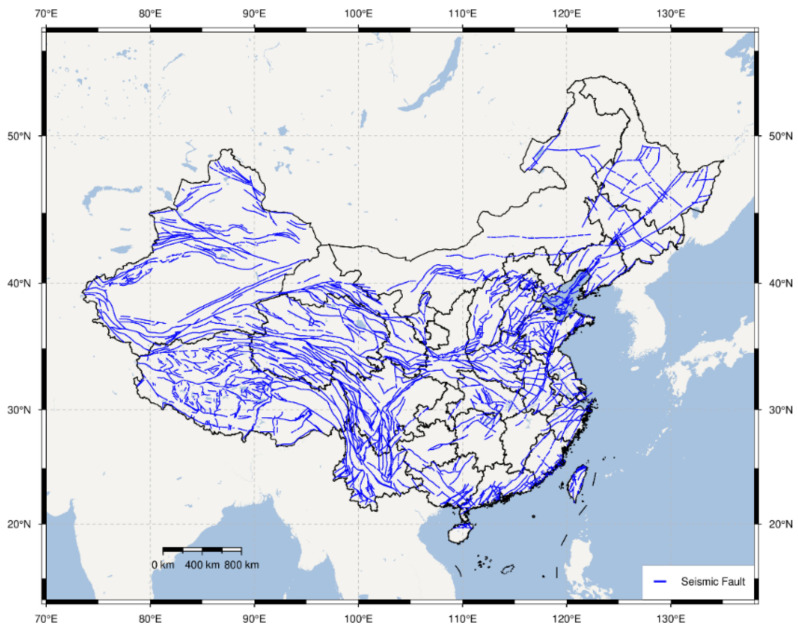
Distribution map of China’s active seismic faults.

**Figure 7:**
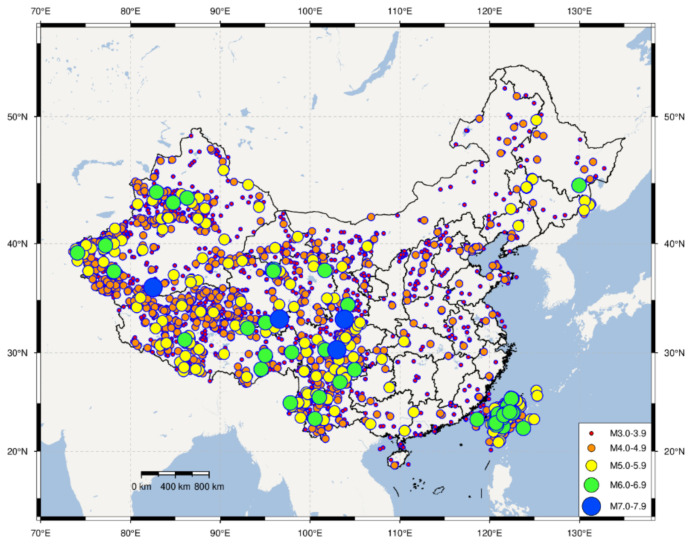
Historical seismic event distribution in China during 2009–2020.

**Figure 8:**
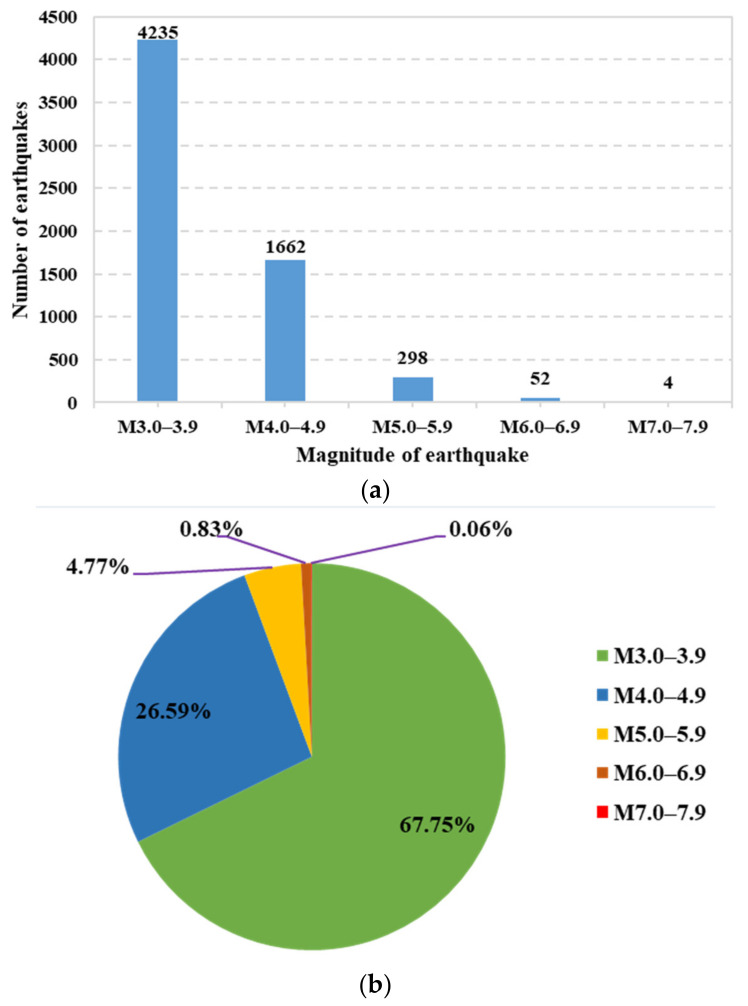
The analysis of the number and proportion of historical earthquakes with five different magnitudes in China 2009–2020. (**a**) The number of the historical earthquakes with five different magnitudes. (**b**) The proportion of the historical earthquakes with five different magnitudes.

China is located in two of the world’s most active seismic belts, namely the circum-Pacific seismic belt and the Eurasian seismic belt. Seismic activities in China are characterized by frequent occurrence, high intensity, shallow source, and wide distribution. According to the data from the China Earthquake Networks Center (CENC) and National Earthquake Data Center (NEDC), the distribution map of China’s active seismic faults and the historical seismic events in China during 2009–2020 are analyzed and depicted in Figure 6 and Figure 7. As seen in Figure 6, active seismic faults in China are numerous and widely distributed. Moreover, earthquakes in China during 2009–2020 with a magnitude larger than M3 (Magnitude level: 3) are counted and displayed on the China map, as seen in Figure 7. Further, the statistical analysis of the historical seismic events is conducted and shown in Figure 8. It can be found that the numbers (proportions) of earthquakes with magnitudes among M3.0–3.9, M4.0–4.9, M5.0–5.9, M6.0–6.9, and M7.0–7.9 are 4235 (67.75%), 1662 (26.59%), 298 (4.77%), 52 (0.83%), and 4 (0.06%), respectively, in the past twelve years. The above analysis indicates that buildings of many cities in China are potentially subjected to high risk from earthquakes. As a result, cost-effective building safety monitoring and its extensive application currently draw more attention from researchers and disaster prevention institutes.

**Figure 9:**
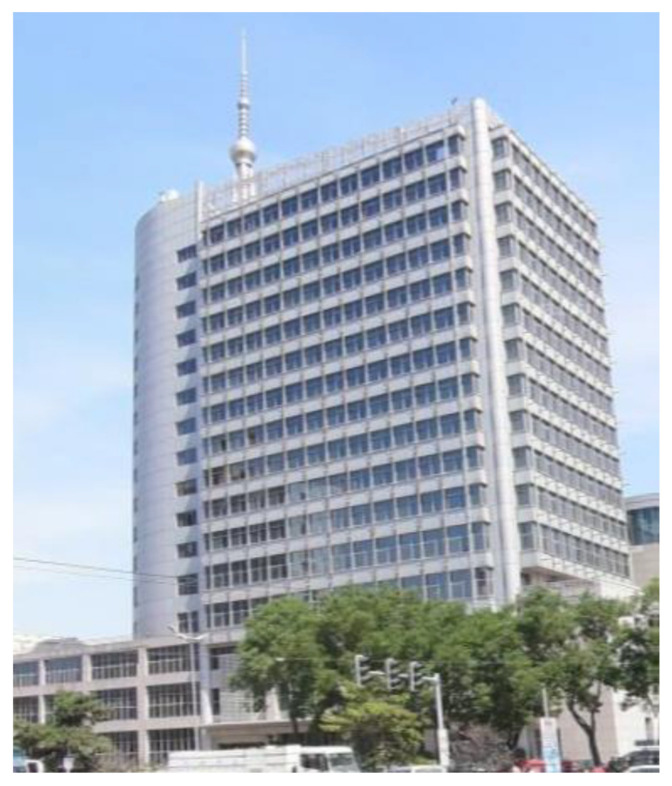
The Changping Guangdian mansion in Beijing city.

According to the statistical data of active seismic faults and seismic distribution in Figure 6, Figure 7 and Figure 8, it is noted that Beijing is located in an earthquake-prone area and is often affected by earthquakes. Beijing is the capital city of China, and is also a political, economic, and cultural center, which is characterized by a developed economy, a dense population, extensive development of underground space, and many aging or even historical buildings. The safety of aging buildings under seismic risk is a critical issue for the public, and thus the building performance assessment after an earthquake event is especially important. To study the building safety monitoring with cost-effective MEMS accelerometers for rapid after-earthquake assessment, an in-service public building, Changping Guangdian Mansion, located in the earthquake-prone area in Beijing is selected (see Figure 9). The main building of this mansion is a 16-floor reinforced concrete frame-shear wall structure building with a height of 48 m aboveground with a basement. The building was first used in early 2005.

### 2.3. Establishment of Building Safety Monitoring System

To construct a safety monitoring system for the building, a deployment scheme for sensor placement, data acquisition, and transmission is developed according to the following principle. As the maximum displacement of the building top and the maximum IDR profile are adopted as the building condition assessment indexes in this study, a limited number of sensors is required for building safety assessment. The MEMS sensors should be appropriately placed to measure the information necessary for computing the building condition assessment indexes. Generally, the sensors should be evenly distributed in a straight line along the height of the building, and the sensors should be preferentially installed on certain important floors, such as the top of the building, reinforced floors of a tall building, and certain floors containing vulnerable structures, etc. The more sensors there are along the straight line of the building, the higher the obtained spatial resolution of the IDR profile.

**Figure 10:**
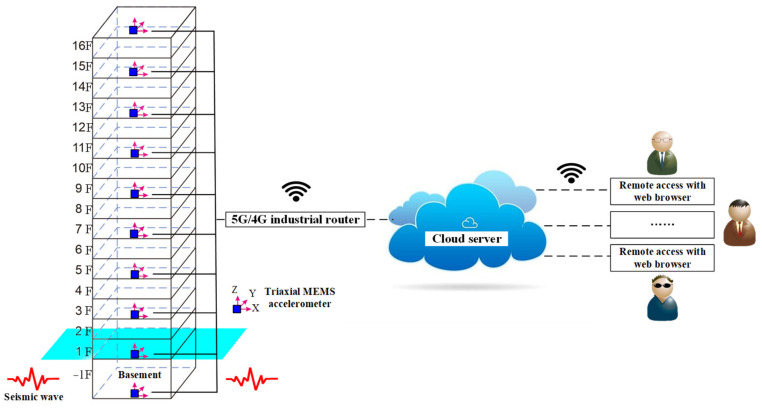
Schematic diagram of the building safety monitoring system.

According to the principle mentioned above, ten triaxial MEMS accelerometers are installed on the western walls of weak electricity rooms to measure the structural responses in X-, Y-, and Z-directions (West–East, South–North, and vertical directions). As shown in Figure 10, the accelerometers are placed in the basement and the 1st, 3rd, 5th, 7th, 9th, 11th, 13th, 15th, and the top of the Guangdian building. Considering data synchronization, the ten sensors used for building-safety monitoring are connected to an industrial router by wire. Then, the collected data are transmitted to cloud servers through a 5G/4G wireless network. Thereafter, all the authorized users can gain remote access to the cloud server to observe the signals and condition assessment results of the building.

## 3. Methodology for Rapid after-Earthquake Building Safety Assessment

Ground motions induced by earthquakes can lead to abrupt or cumulative damage to buildings, and thus a rapid assessment method is developed in this section. The proposed rapid after-earthquake building-safety assessment will adopt the maximum displacement and the maximum IDR of a building as safety indexes. However, the in-situ measurement may encounter missing data problems, and the displacement responses cannot be directly measured but can be indirectly estimated. Therefore, Section 3.1, Section 3.2 and Section 3.3 will introduce the methods for missing data reconstruction, displacement estimation by an integral of acceleration, and the calculation of IDR, respectively.

### 3.1. Method for Fast Missing Data Reconstruction

Missing data is a typical problem in the data acquisition process during structural condition monitoring due to transmission error and sensor fault [52]. Incomplete monitoring data can cause false analyses and missed detections of events, leading to inappropriate decisions made for arranging maintenance and contingency measures. Therefore, the accurate reconstruction of missing data is of great importance, especially for emergencies such as the case of an earthquake. Missing data is a common problem due to the use of wireless transmission, and it happens inadvertently. In this study, the incomplete monitoring data may result in the inability to correctly calculate the maximum displacement and IDR profile, which are the indexes for assessing the after-earthquake safety of a building. In other words, missing data could lead to an incorrect assessment of building safety conditions and false alarms.

Many existing studies have focused on data reconstruction through mean and median values, or simple interpolation methods, which are characterized by easy implementation but low accuracy [53]. To overcome this drawback, researchers are devoted to the study of efficient and accurate missing data reconstruction algorithms [54,55,56,57,58]. The matrix-decomposition-based data reconstruction method (e.g., low-rank matrix/tensor completion) [55] is one of the widely advocated algorithms due to the direct expression of the relationship between time series and spatial locations. Tensor completion methods can be used in different fields to solve the missing data problem and are generally used for image completion and traffic data imputation [59,60,61,62,63,64]. However, investigations on their application to the data imputation of three-axial coupled structural responses of buildings under seismic excitation for building safety assessment are rarely reported.

To reach high-accuracy data reconstruction, we propose the use of the following low-rank tensor completion method with a fast-solving algorithm. The well-known optimization problem for low-rank tensor completion can be formulated as

(1) $$\begin{array}{l} \arg\underset{\chi }{\min}:∥\chi {∥}_{*}:=\sum_{i=1}^{n}\alpha_{i}∥\chi_{\left(i\right)}{∥}_{*}\\ s.t.:\chi_{\Omega }=T_{\Omega } \end{array}$$

where T is an *n*-mode (dimensional) input tensor constituted by the measured dynamic responses with missing entries, χ is an estimated *n*-mode tensor after missing data reconstruction of T, subscript Ω denotes the subset of T and χ, which does not need to be reconstructed, and $\alpha_{i}$ is the weighting constant satisfying $\alpha_{i}\geq 0$ and $\sum_{i=1}^{n}\alpha_{i}$ = 1.

The difficulty in efficiently solving the tensor-trace-norm-related minimization problem in Equation (1) is that multiple dependent non-smooth terms exist in the objective function. Nesterov [65] proposed a general method to solve a non-smooth optimization problem. The smooth version of the minimization problem in Equation (1) can be expressed as

(2) $$\begin{array}{l} \arg\underset{\chi }{\min}:\sum_{i=1}^{n}\max\alpha_{i}\langle \chi ,y_{i}\rangle -\frac{\mu_{i}}{2}{\left\|y_{i}\right\|}_{F}^{2}\\ s.t.:\chi_{\Omega }=T_{\Omega } \end{array}$$

where $\mu_{i}$ is a positive constant and $y_{i}$ is a dual variable. For solving this smooth minimization problem above, a fast low-rank tensor completion algorithm is to be employed. The comparison among the tensor completion algorithms, including Tucker-, Parafac-, and SVD-based methods, SiLRTC, HaLRTC, and FaLRTC was conducted, and the results showed that (i) the last three algorithms could work with a small number of samples and fill larger missing regions, (ii) the HaLRTC and FaLRTC methods exhibited better reconstruction performance than the others, and (iii) between FaLRTC and HaLRTC, the former proved to be more efficient [59]. Therefore, the FaLRTC algorithm is adopted in this paper for achieving good reconstruction performance with satisfactory computational efficiency. In this study, the tensor is constructed by placing the acceleration response matrices along the X-, Y-, and Z-directions (West–East, South–North, and vertical directions) into its first, second, and third layers, respectively, such that the missing data along three directions can be simultaneously estimated by solving Equation (2). Therefore, this paper takes advantage of the FaLRTC method in terms of its direct exploitation of the relationship between time series and spatial locations and its fast-convergency property for the three-axial coupled missing seismic responses reconstruction.

### 3.2. Method for Displacement Estimation

Accelerometers are widely used for on-site structural health monitoring of civil structures. Compared with velocity or displacement responses, acceleration responses can be easily measured by less expensive sensors and the acceleration signals have a high signal-to-noise ratio. Nonetheless, displacement is necessary for rapid after-earthquake assessment of a building, because it is directly related to elastic or plastic deformation and structural damage, and the thresholds of displacement rather than acceleration are clearly defined in current seismic codes. After missing data reconstruction, the displacement can be estimated by the integral of acceleration data. To avoid error accumulation during the double integral process of the time-domain integral method and to improve computational efficiency, the frequency-domain integral method is adopted in this study for an accurate and fast integral. Specifically, the velocity and displacement responses are estimated respectively using the following equations.

(3) $$\dot{x}(l)=\sum_{k=1}^{N-1}\frac{1}{j2\pi k\Delta f}H(k)\ddot{X}(k)e^{j2\pi kl/N}$$

(4) $$x(l)=\sum_{k=1}^{N-1}-\frac{1}{{\left(2\pi k\Delta f\right)}^{2}}H(k)\ddot{X}(k)e^{j2\pi kl/N}$$

with

(5) $$H(k)=\left\{\begin{array}{l} 1\;(f_{d}\leq k\Delta f\leq f_{u})\\ 0\;(\mathrm{others}) \end{array}\right.$$

where $\ddot{x}(l)$, $\dot{x}(l)$, and x(l) are the vectors of acceleration, velocity, and displacement responses, $\ddot{X}(k)$ is the Fourier transform of $\ddot{x}(l)$, k and l are sequence number in time and frequency domain, Δf is frequency resolution, H(k) is a filter function, $f_{d}$ and $f_{u}$ are lower cut-off frequency and upper cut-off frequency, and kΔf falls in the interval between the lower and upper cutoff frequencies. The estimated displacement responses are used to calculate the maximum displacement of the top of the building and the maximum IDR profile for the building safety assessment in Section 4.2.

### 3.3. Method for Inter-Story Drift Ratio Calculation

Advanced damage identification techniques with complex algorithms are often difficult for fast assessment. IDR is one of the most widely used and effective indicators for the performance assessment of tall buildings, which can fulfill the fast and effective assessment requirement. Thus, IDR is employed in this study for structural condition and damage extent assessment. It is derived from the definition of inter-story drift, which is defined as the relative translational displacement difference between two consecutive floors in current seismic codes [66,67]. 

**Figure 11:**
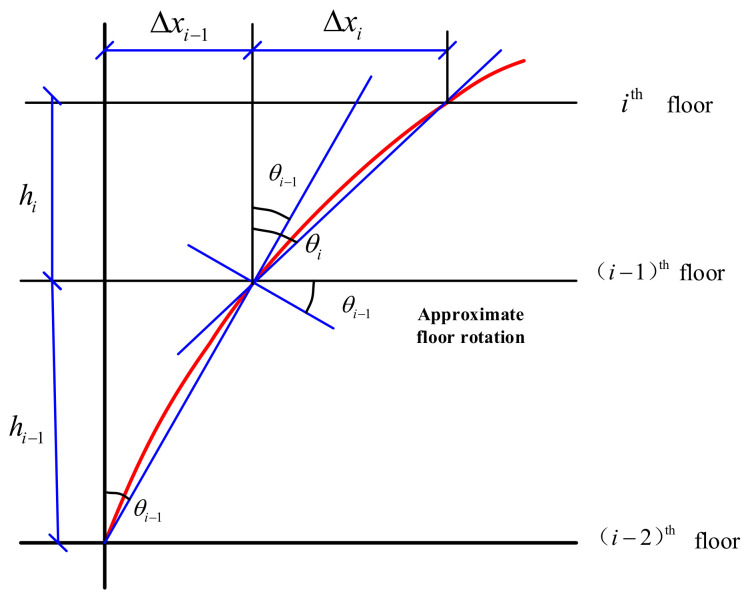
The secant method for calculating IDR.

The inter-story drift consists of the inter-story shear drift and flexural drift induced by the vertical members. To evaluate the safety condition of a building, the IDR can be calculated [68] as:

(6) $$\theta_{i}=\frac{\Delta x_{i}}{h_{i}}=\frac{x_{i}-x_{i-1}}{h_{i}}$$

where $\theta_{i}$ is the IDR; $x_{i}(x_{i-1})$ is the horizontal displacement of the $i^{\mathrm{th}}(i-1^{\mathrm{th}})$ floor; $h_{i}$ is the story height between the $i^{\mathrm{th}}$ and $i-1^{\mathrm{th}}$ floor. To achieve an earlier warning, the IDR computation in Equation (6) uses a conservative calculation method, i.e., the secant method [69] as shown in Figure 11.

The building-safety monitoring system with cost-effective MEMS accelerometers for rapid after-earthquake assessment should be able to detect whether the building structure remains in a safe state and release alarms immediately when the structural responses exceed the predefined safety thresholds. This is essential to prevent fatalities and economic loss. To describe the structural safety conditions and send out different levels of alarms, proper safety criteria corresponding to different structural health states should be predefined. The building safety conditions are divided into three levels according to the current seismic codes, and the proposed MEMS-sensor-based approach can use the IDR index to classify building safety conditions into elastic, elasto-plastic, or plastic deformation states. The building safety level is assessed according to the calculated IDR and the threshold values for IDR are listed in Table 2. Taking the building with the R.C. frame-shear wall structure type in this paper as an example, if the IDR falls in the intervals IDR < 1/800 or 1/800 ≤ IDR < 1/100, it implies elastic deformation or elasto-plastic deformation occurs, followed by an “immediate occupancy” or “occupancy after repair” contingency measure. If the IDR ≥ 1/100, it implies severe damage occurs and “collapse prevention” should be taken. The 1/800 and 1/100 are the alarm thresholds of IDR adopted in this study, which are in accordance with the Chinese code GB50011-2010 for Seismic Design of Buildings and the Chinese code JGJ3-2010 Technical Specification for Concrete Structures of Tall Building [67,68,70].

### 3.4. Procedure of Rapid After-Earthquake Assessment

**Figure 12:**
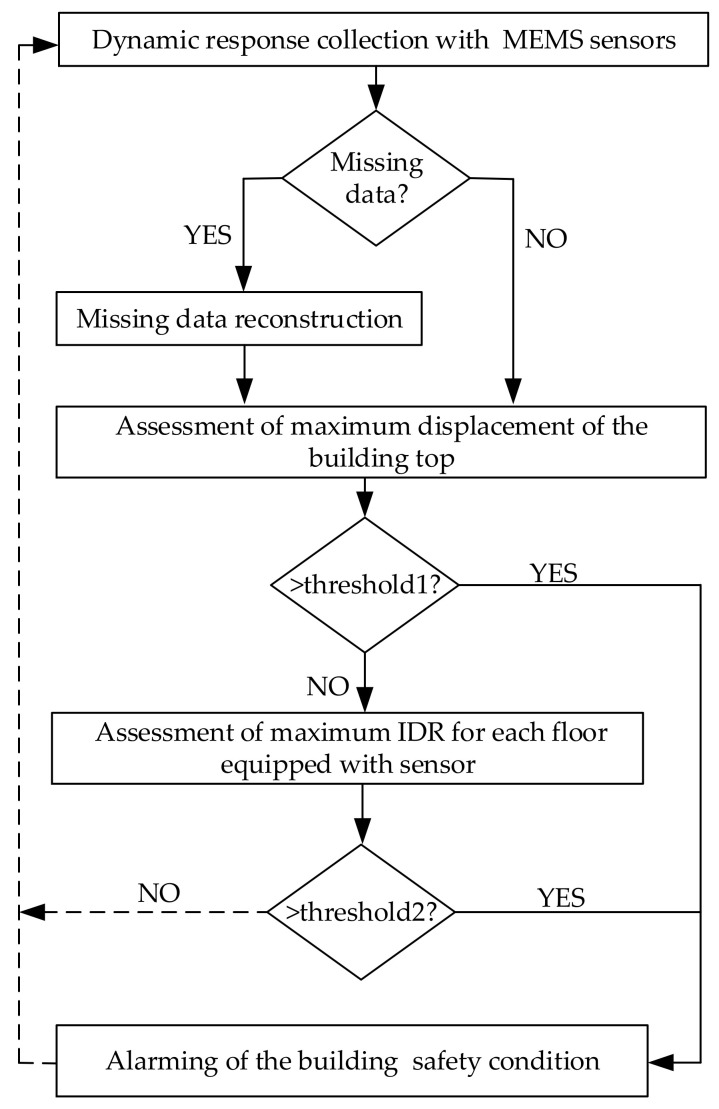
Flowchart of the proposed rapid after-earthquake building safety assessment method.

The flowchart of the proposed rapid after-earthquake building safety assessment scheme based on the use of cost-effective MEMS accelerometers is shown in Figure 12. It includes the following steps:

**Step1:** Collect the dynamic acceleration responses by using the cost-effective MEMS accelerometers installed in the selected building.

**Step2:** Check if the missing data problem happens to the measured acceleration responses and reconstruct the missing data by using the fast reconstruction approach (Equations (1) and (2)).

**Step3:** Assess the maximum displacement of the building top using a radar map after the frequency-domain integral of acceleration and trigger the alarm once the maximum displacement exceeds the predefined threshold.

**Step4:** Assess the maximum IDR for all floors equipped with MEMS accelerometers and trigger the alarm once the maximum IDR exceeds the predefined threshold.

**Step5:** Repeat Step 1 to Step 4 for continuous building-safety monitoring and condition assessment.

## 4. Validation of Building Safety Assessment Method

The rapid after-earthquake assessment method was carried out after the implementation of the proposed SHM monitoring system in Figure 10. The building-safety monitoring system detected several seismic events of different magnitudes. Dynamic seismic responses were successfully captured by the proposed cost-effective structural health-monitoring system equipped with the adopted MEMS sensors during seismic events. One of the typical events is the 2019 Tangshan earthquake with a magnitude of 4.5, the hypocenter of which was located at 10 km in depth, 39.3° in latitude and 118.04° in longitude, and 184.4 km away from the Changping Guangdian Mansion in Beijing city. The selected event has a typical data-missing scenario for the validation of the building safety assessment scheme. In this section, the fast tensor-completion algorithm was employed for missing data reconstruction of the three-dimensional monitoring responses. The displacement responses were estimated by the fast integral of acceleration after missing data estimation. On this basis, a rapid after-earthquake assessment of the building was performed by assessing the maximum displacement of the top of the building and the maximum IDR profile of the whole building.

### 4.1. Missing Data Reconstruction

**Figure 13:**
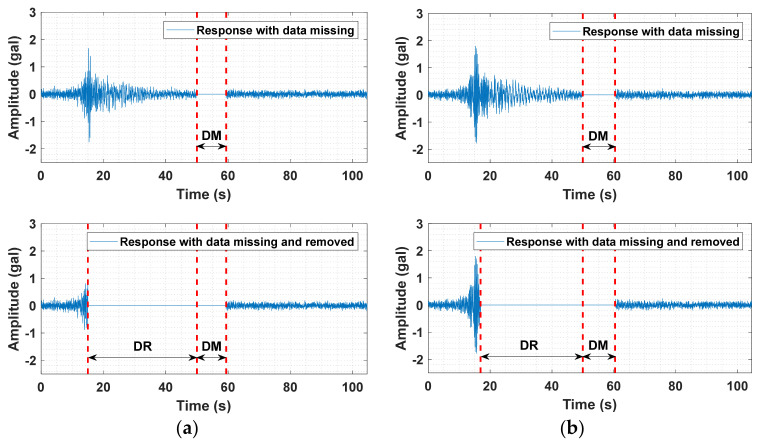

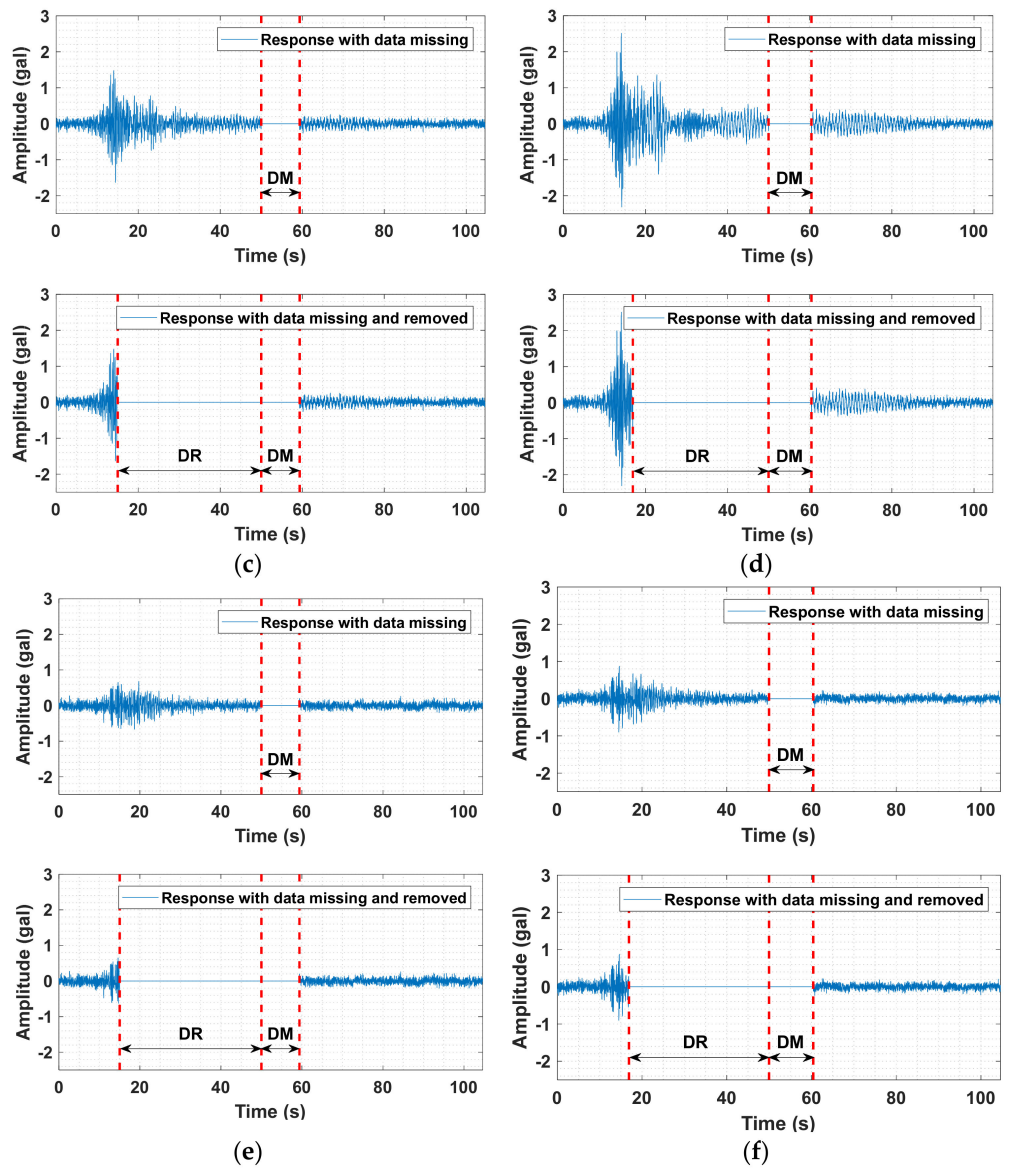
Responses with missing data (DM) and data artificially removed (DR). (**a**) X-axial vibration of the third floor. (**b**) X-axial vibration of the seventh floor. (**c**) Y-axial vibration of the third floor. (**d**) Y-axial vibration of the seventh floor. (**e**) Z-axial vibration of the third floor. (**f**) Z-axial vibration of the seventh floor.

Missing data occurred in the data acquisition process of the third and seventh floors during the 2019 Tangshan earthquake (Mag. = 4.5). Specifically, the original monitoring responses with missing data are displayed in Figure 13. There are six graphs in this figure. The graphs in (a), (c), and (e) show the responses of the third floor, and those of the seventh floor are depicted in the right-side column of Figure 13. In each graph, “DM” in the upper and lower subplots of each graph represents the part with missing data, and “DR” in the lower subplot of each graph stands for the part with data artificially removed. The target is to reconstruct the missing data, while the DR part is created for validating data reconstruction performance by comparing the original response with the reconstructed response. The absent data, including the missing and removed data, are to be subsequently reconstructed.

**Figure 14:**
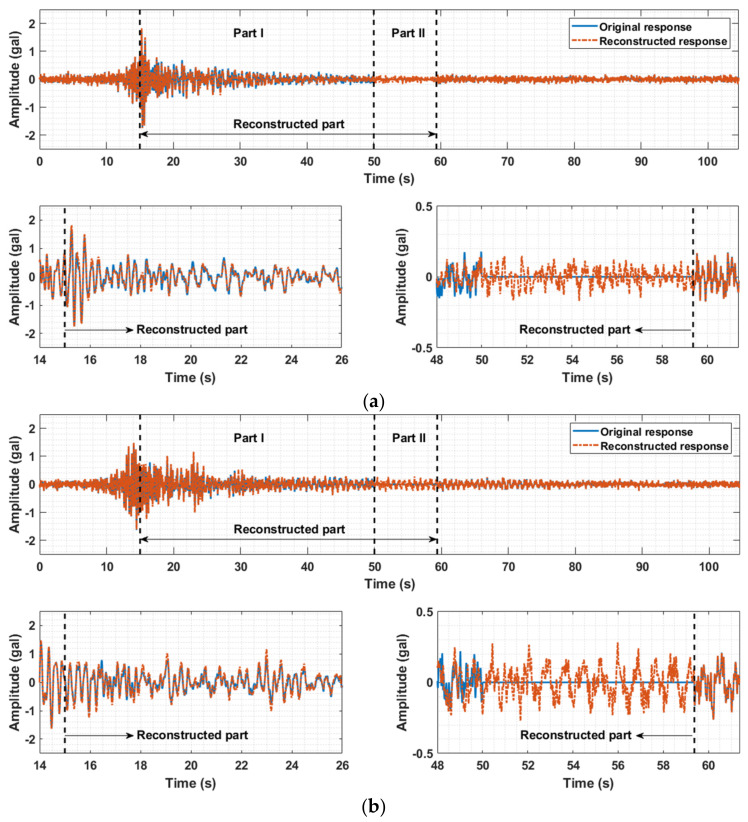

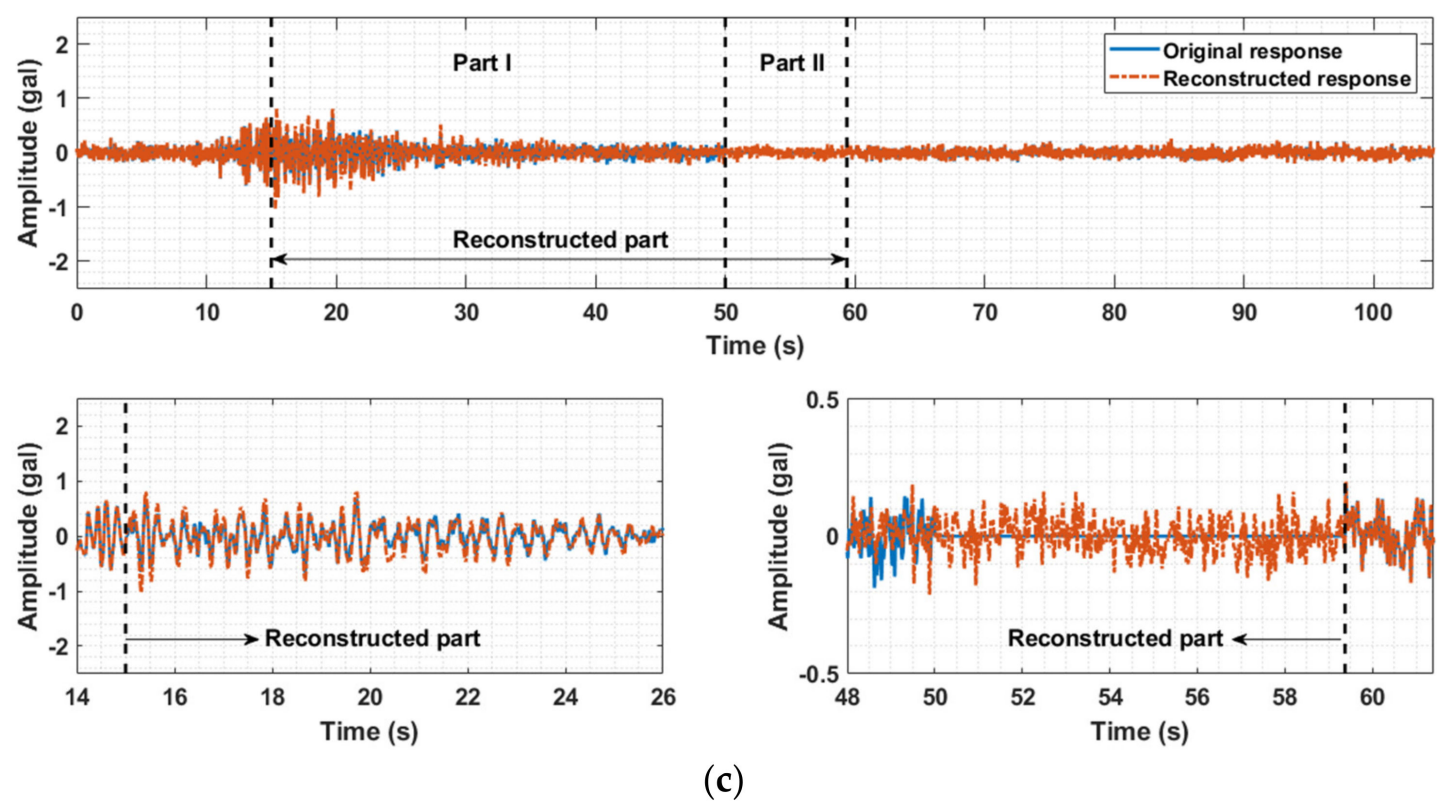
Comparisons between original and reconstructed responses of the third floor. (**a**) X-axial acceleration response of the third floor. (**b**) Y-axial acceleration response of the third floor. (**c**) Z-axial acceleration response of the third floor.

**Figure 15:**
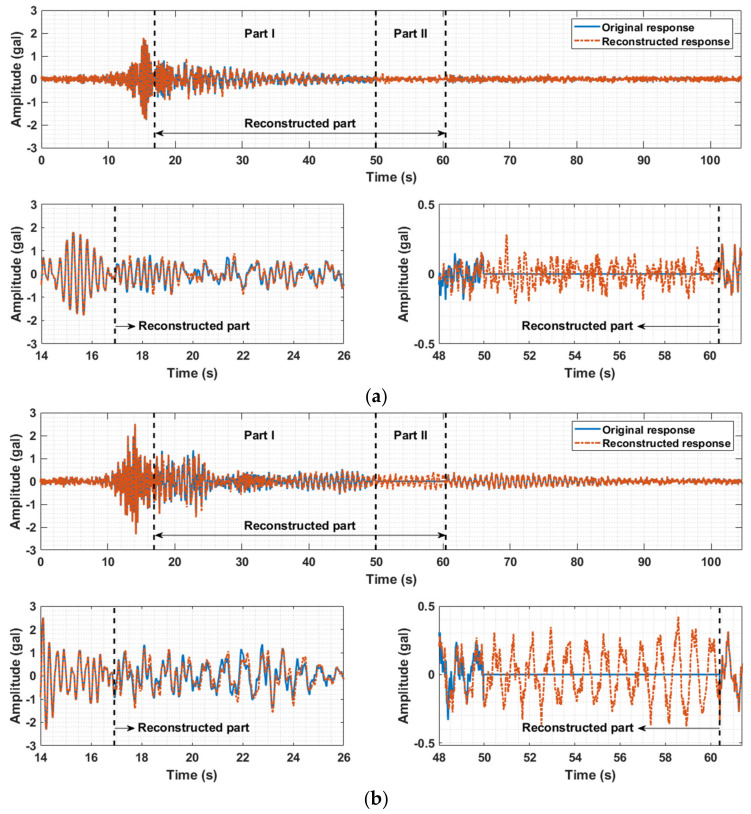

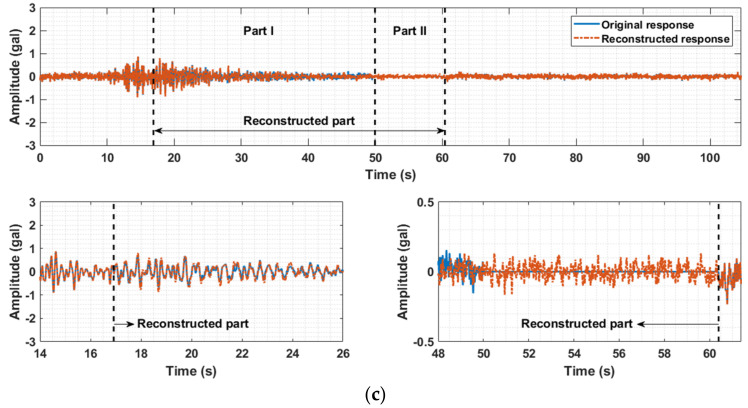
Comparisons between original and reconstructed responses of the seventh floor. (**a**) X-axial acceleration response of the seventh floor. (**b**) Y-axial acceleration response of the seventh floor. (**c**) Z-axial acceleration response of the seventh floor.

To reconstruct the missing and removed data of the measured acceleration responses in the third and seventh floors, the fast low-rank tensor completion method introduced in Section 3.1 was employed. The measured acceleration responses of the ten floors are placed into a three-layer tensor in which the first, second, and third layers are filled with the acceleration data matrices along the X-, Y-, and Z-directions (West–East, South–North, and vertical directions), respectively, to fully capitalize on the temporal-spatial information and simultaneously estimate the triaxial absent data of the two floors. Figure 14 shows the comparison between the original and reconstructed responses of the third floor, and Figure 15 illustrates those of the seventh floor. The three graphs (a)–(c) in Figure 14 and Figure 15 correspond to the triaxial acceleration responses along the X-, Y-, and Z-directions. In each graph, there are three subplots, with the upper subplot showing the entire information and the two lower subplots illustrating detailed information of Part I (the reconstruction of DR part) and Part II (the reconstruction of DM part) in the upper subplot. For the third floor, the upper subplots in Figure 14a–c show both the original measured data (blue line) and the reconstruction data (orange line). The left-side lower subplot displays the enlarged picture of a segment in Part I (the segment between 15 s and 26 s), and it is noted that the reconstructed responses for the artificially removed data highly agree with the original data. This proves the effectiveness of the missing data reconstruction method. In the right-side lower subplot, both the reconstructed values of removed data in Part I (the segment between 48 s and 50 s) and the reconstructed values of missing data in Part II (the segment between 50 s and 59.5 s) are enlarged and displayed together. The reconstructed responses in Part II also exhibit high consistency with the original measured data. For the seventh floor, similar results can be drawn from the observation of Figure 15. A good reconstruction performance is witnessed in both Figure 14 and Figure 15, which illustrate the original responses of two different floors with missing practical data and artificially removed data and their reconstructed responses, and it is noted that the data imputation performance in the case where the peak is missing is also exhibited. The missing peak is obviously shown in Figure 13a and included in part of the artificially removed data. Accordingly, it can be found in Figure 14a that the reconstructed response (orange line in Part I) agrees well with the artificially removed data (blue line in Part I), demonstrating good data reconstruction performance in the case where the region of missing data includes the peak of the response.

### 4.2. Structural Safety Assessment

**Figure 16:**
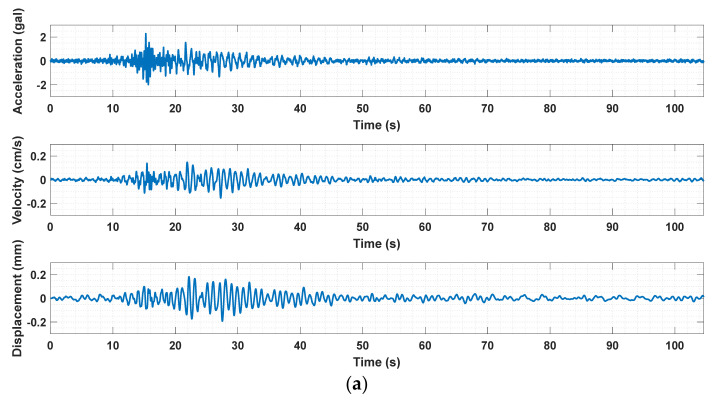

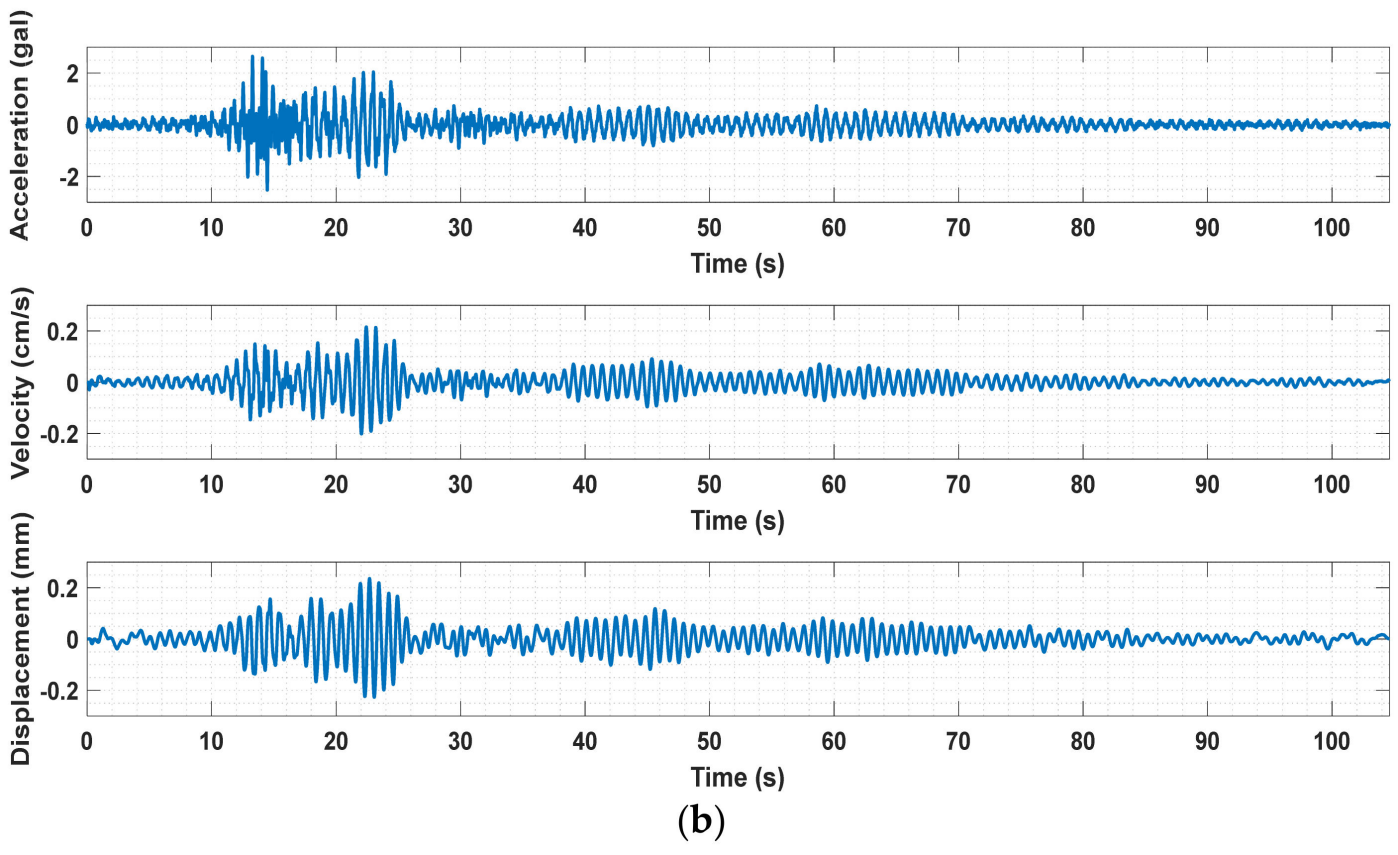
The displacement obtained by the frequency-domain integral of acceleration responses. (**a**) The X-axial velocity and displacement at the building top. (**b**) The Y-axial velocity and displacement at the building top.

To conduct the rapid after-earthquake assessment of this building, the lateral displacement responses were firstly obtained by calculating the integral of the acceleration responses using Equation (4) after missing data reconstruction. As an example, Figure 16 shows the estimated X-axial and Y-axial velocities and displacements at the top of the building subjected to the 2019 Tangshan earthquake.

**Figure 17:**
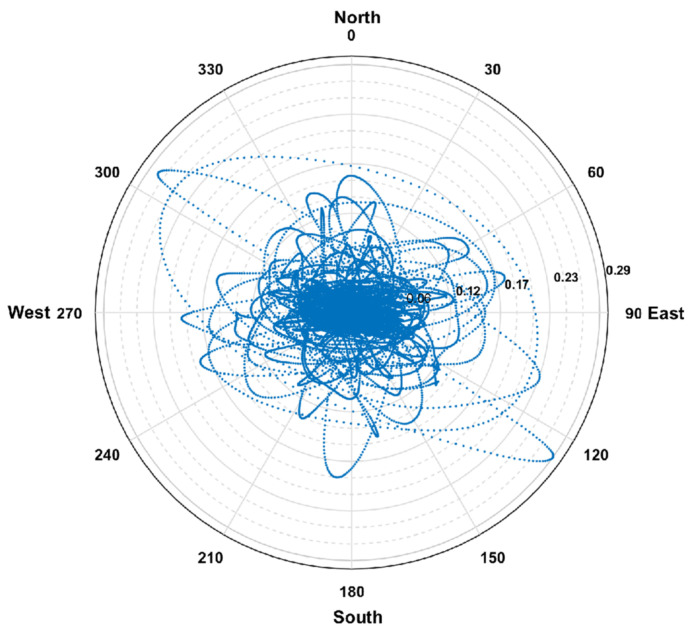
Radar map at the top of the building under the ground excitation of 2019 Tangshan earthquake.

To intuitively observe the motion trail of the top of the building, a radar map was used to illustrate the direction and amplitude of the tall building monitored. The radar map can be drawn using both the X-axial and Y-axial displacements of the measurement point as depicted in Figure 16. For the specific case study of the 2019 Tangshan earthquake, the radar map for the motion trail of the building top is shown in Figure 17. Once the maximum motion exceeds the safety threshold, the monitored building may suffer from structural damage, and alarms should be triggered. For the 2019 Tangshan earthquake, it can be found from Figure 17 that the maximum structural displacement occurs along the direction between 120 degrees and 150 degrees with an amplitude of 0.29 mm. The motion-trail threshold for rough assessment was calculated using $x_{\lim}=\theta_{\lim}H$, where $\theta_{\lim}$ is the limit of IDR according to seismic design codes. The threshold for elastic IDR of the R.C. frame-shear wall building in Table 2 is $\theta_{\lim}=1/800$ and the height between the top floor and the ground is H=48m; thus, the maximum displacement threshold for the motion trail of the top floor is 60 mm. The maximum displacement in the motion trail of the building top is 0.29 mm, which is much smaller than the displacement threshold 60 mm. Therefore, the building is probably in a safe condition after the earthquake, according to the rough assessment using a radar map and the displacement threshold. It should be noted that this is a simple and easy approach for a rough and quick assessment, and it is applicable to the situation in which there is only one MEMS sensor installed in the building and the sensor is installed on the top of the building. Besides, if the number of sensors is very limited but still more than one, this method can be used to roughly evaluate the maximum displacement of the few floors equipped with the MEMS sensors after H is changed to the height between the instrument floors and the ground.

**Figure 18:**
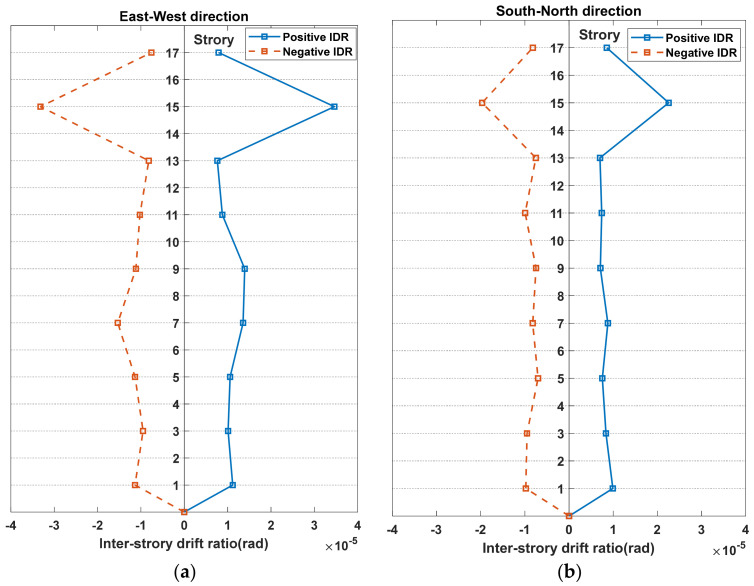
Maximum IDR under the ground excitations of 2019 Tangshan earthquake. (**a**) The X-axial IDR. (**b**) The Y-axial IDR.

After the above rough assessment, further assessment using the IDR was conducted to confirm the building-safety condition. The IDR of the building structure subjected to the 2019 Tangshan earthquake was calculated using Equation (6) and applied to evaluate the safety condition of the building after the earthquake. For all the floors equipped with MEMS sensors, the maximum IDR along the East–West direction and South–North directions of the building were computed and are illustrated in Figure 18. It is noted that the maximum values in the IDR curve for the East–West direction in Figure 18a are $-3.32\times 10^{-5}$ and $3.46\times 10^{-5}$, while those for the South–North direction in Figure 18b are $-1.97\times 10^{-5}$ and $2.25\times 10^{-5}$. All the maximum IDRs are below the minimal predefined limit listed in Table 2, which indicates that the building-safety condition is satisfactory. As confirmed by human inspection after the earthquake, these assessment results coincide with the actual situation of the building.

The main contribution of this paper is the proposal and application study of the rapid after-earthquake assessment scheme for building-safety assessment using cost-effective MEMS accelerometers. The proposed scheme systematically consists of fast missing data reconstruction, fast displacement estimation based on the frequency-domain integral of the acceleration response, and maximum displacement and maximum IDR-based assessment. The fast missing data reconstruction method (FaLRTC) serves as one functional component in the scheme to solve the missing data problem in the practical scenario. This study examined its applicability to this problem using an in-service building subjected to real earthquakes and demonstrated its effectiveness by reconstructing artificially removed data and missing practical data. On this basis, the maximum displacement and maximum IDR profile were then obtained and successfully applied to the building safety assessment.

## 5. Discussion

**Figure 19:**
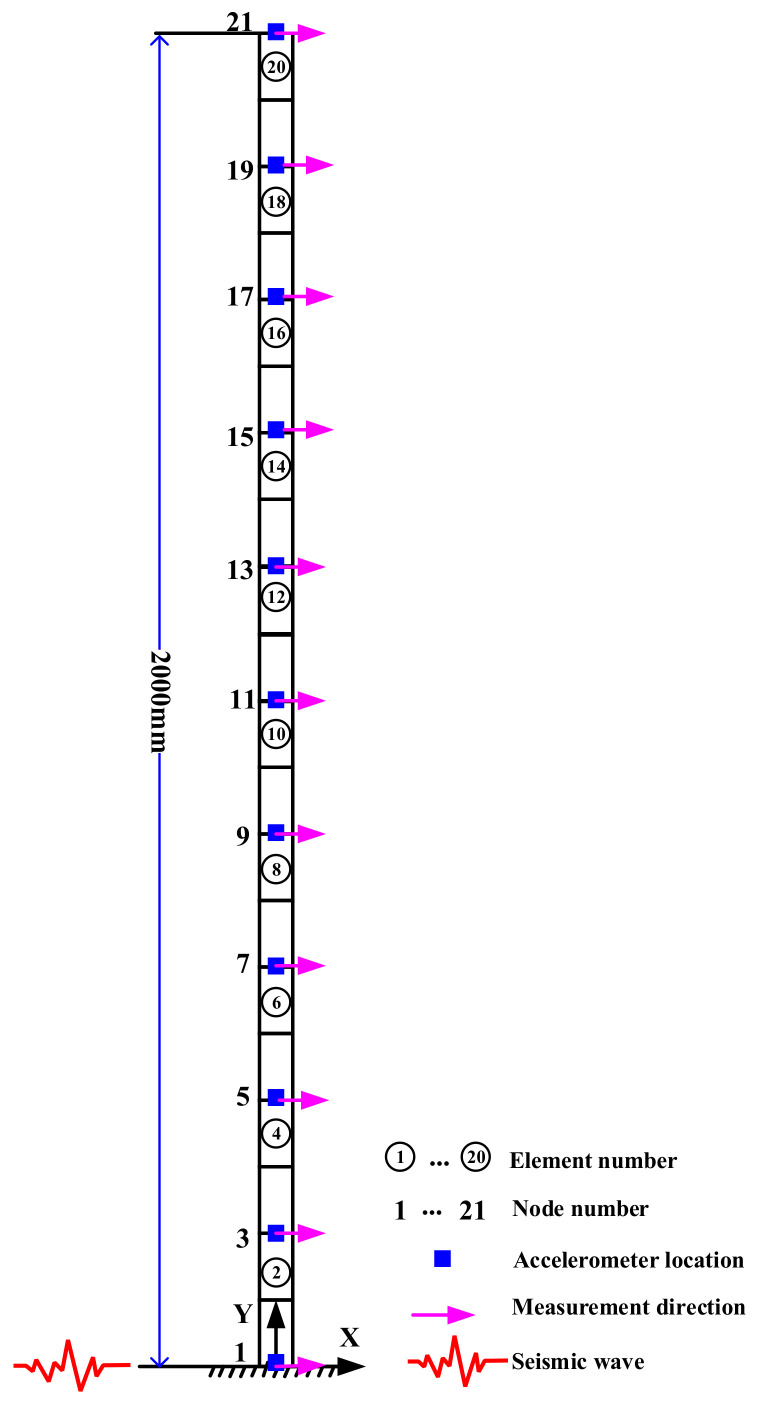
The finite element model of a 2D cantilever beam structure.

**Figure 20:**
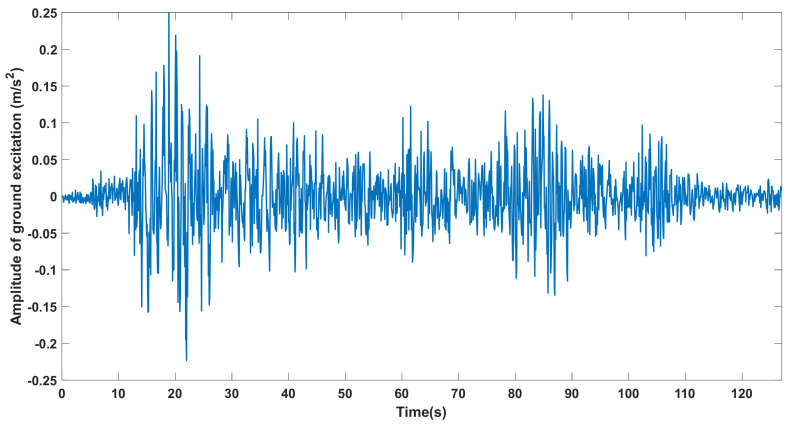
The scaled seismic wave of the 1985 Michoacán Earthquake (PGA = 0.025 g).

A numerical study was conducted using a 2D cantilever steel beam model to further discuss the accuracy and convergency rate of the missing data reconstruction method and demonstrate the relationship between the proposed IDR index and the structural damage of a building. As shown in Figure 19, the finite element model of the cantilever beam consists of 21 nodes and 20 equal-length beam elements. The structural damping is assumed to be Rayleigh damping with the first two damping ratios $\xi_{1}=0.01$ and $\xi_{2}=0.01$. The Young’s elastic modulus and density are, respectively, 210 GPa and 7850 kg/m^3^. The cantilever beam model is subjected to ground excitation in the x-direction. The scaled seismic wave of the 1985 Michoacán Earthquake was employed as the ground excitation for the numerical study. Considering the moderate earthquake design criterion in the Chinese Seismic Design Code [67], the peak ground acceleration (PGA) of the ground excitation was scaled to 0.025 g (0.25 m/s^2^) and the scaled seismic excitation is shown in Figure 20. Eleven accelerometers were evenly installed on the beam model to measure the x-direction structural acceleration responses under seismic excitation.

**Figure 21:**
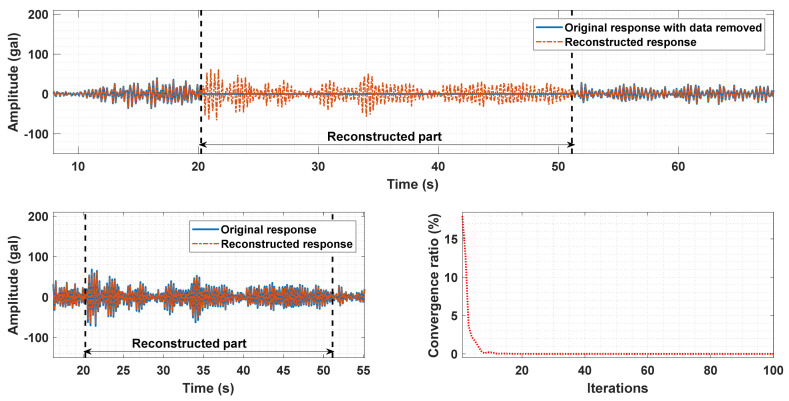
Data reconstruction performance in the intact scenario of the beam structure.

**Figure 22:**
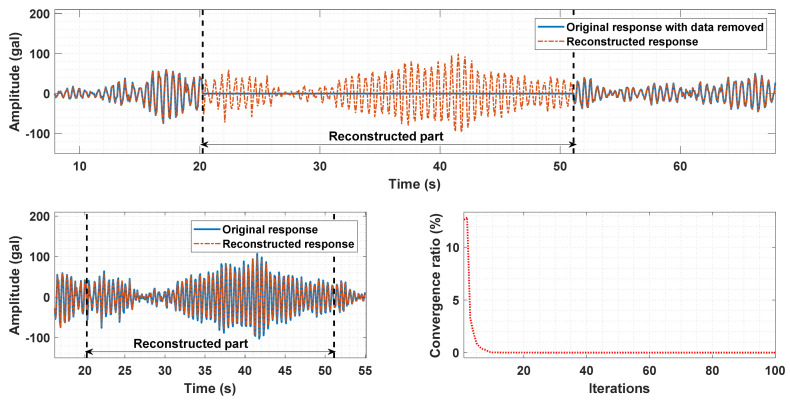
Data reconstruction performance in the damaged scenario of the beam structure.

The missing data reconstruction performance was assessed in two cases. In the first case, the beam structure was in intact state (intact scenario), and in the second case, it was assumed that beam elements 1, 2, and 3 have an 80% stiffness reduction (damaged scenario). In both cases, a data segment of the acceleration response at Node 21 was removed and the data reconstruction method described in Section 3.1 was used to estimate the removed data. The data reconstruction performance in the intact and damaged scenarios are shown in Figure 21 and Figure 22, respectively. For the intact scenario, the response with the removed segment (blue line) and the response after reconstructing the removed data (orange line) are depicted in the upper subplot of Figure 21. The comparison between the original segment and the reconstructed segment is illustrated in the left-side lower subplot of Figure 21. The reconstruction error calculated by $e=\left\|\chi -T_{0}\right\|/\left\|T_{0}\right\|\times 100\%$ is 3.7%, in which $T_{0}$ is the tensor of the eleven original responses and χ is the tensor of the responses with the reconstructed segment. The convergence curve calculated by $r=\left\|\chi_{k+1}-\chi_{k}\right\|/\left\|T\right\|\times 100\%$ is displayed in the right-side lower subplot of Figure 21, in which *k* represents the iteration number and T is the tensor of responses with the data removed. Fast convergency of the data imputation algorithm is witnessed in this subplot. Similarly, good agreement between the removed data and the reconstructed data in the damaged scenario is also observed in Figure 22. In this scenario, the reconstruction error is 2.9% and fast convergency is also found in the right-side subplot of Figure 22.

**Figure 23:**
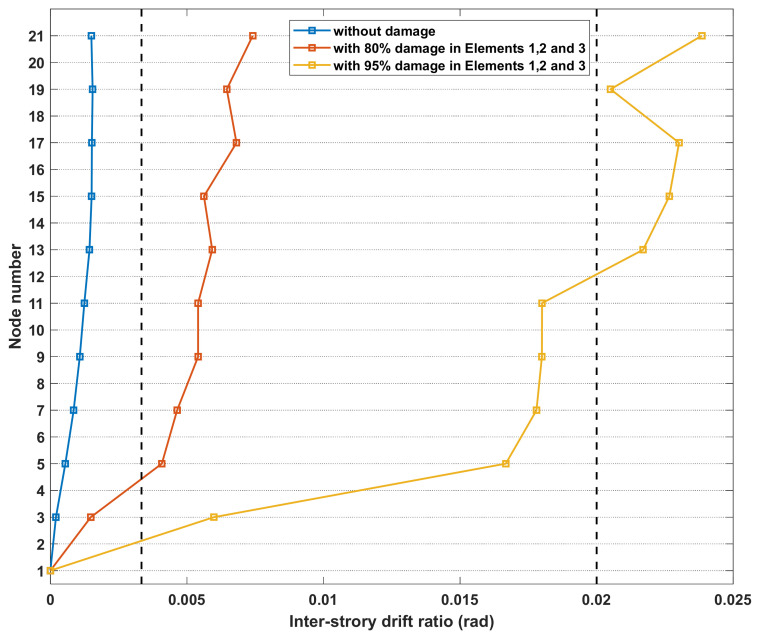
IDRs of the cantilever beam in three scenarios with different damage levels (ground excitation PGA = 0.025 g).

After missing data reconstruction, a structural safety level assessment using the IDR index in Equation (6) and thresholds in Table 2 was conducted for the cantilever beam model. In this numerical study, three different scenarios representing intact, moderate-, and severe-damage states are created by producing a 0%, 80%, or 95% stiffness reduction in the first three elements close to the fixed end suffering from a higher stress concentration. The IDRs of the cantilever beam structure subjected to the scaled seismic wave of the 1985 Michoacán Earthquake in the three scenarios were calculated and are depicted in Figure 23. Under the same seismic excitation, the three maximum IDR profiles (the blue, orange, and yellow curves) are obtained when the beam model has a 0%, 80%, or 95% stiffness reduction in the first three elements. It can be found that the three curves fall into the three different categories according to the thresholds for a steel building structure in Table 2. In the first scenario, the maximum value of the x-direction IDR curve is 0.0016, which is smaller than the threshold (IDR < 1/300) for building safety level 1 and indicates that the structure is in a safe condition. For the second scenario, the maximum value of the x-direction IDR curve is 0.0067, which lies in the range of building safety level 2 with 1/300 ≤ IDR < 1/50 and implies that the structure encounters moderate damage. For the third scenario, the maximum value in the x-direction IDR curve is 0.0268, which reaches building safety level 3 with IDR > 1/50 and thus reveals that severe damage occurred to the structure and collapse-prevention measures should be taken. Therefore, the proposed method can identify the occurrence of structural damage, classify the structural conditions into correct categories, and suggest contingency measures according to the identified safety levels. Although the IDR index cannot identify damage locations or provide quantitative damage extents for structural components, the proposed assessment method can realize the global assessment of the entire building and efficiently classify the building conditions into different safety levels even in missing data situations.

## 6. Conclusions

An application study of cost-effective building-safety monitoring and rapid after-earthquake assessment has been conducted with cost-effective MEMS accelerometers deployed in an in-service building. The sensor parameter analysis revealed the MEMS accelerometers’ low noise level and capability to acquire low-amplitude vibration in the frequency band of interest, which demonstrated their suitability for building monitoring. The adopted cost-effective MEMS accelerometers were installed in the selected building and a cost-effective monitoring system was established. A rapid after-earthquake assessment method was proposed, which consisted of fast missing data reconstruction, fast displacement estimation based on the frequency-domain integral of acceleration response, and maximum displacement and maximum IDR-based assessment. The above three steps formed the rapid assessment scheme that sets different alarm levels in accordance with the safety-related thresholds defined in seismic design codes.

The performance of the assessment scheme was examined by a case study using the in-situ monitoring seismic responses. When the missing data problem occurred for different floors and different time segments in a seismic event, the proposed method can accurately reconstruct the missing data including the missing peak. Thereafter, the displacement responses were obtained by the frequency-domain integral of acceleration. The rough assessment was conducted through a radar map and the displacement threshold, and the three-level safety assessment was further performed via the maximum IDR. The case study demonstrated that the cost-effective monitoring system and the proposed assessment method were able to obtain the building displacement responses under seismic events in a timely manner and provide rapid assessment results to support contingency management. The significance of this study is to enhance the solid connection between laboratory exploration and engineering application of MEMS-based cost-effective building monitoring and rapid after-earthquake assessment.

## Figures and Tables

**Table 1:** Specifications of the selected triaxial MEMS accelerometer.

Parameters	Specifications
Measurement Range	± 2000 mg
Noise Dynamic Range	>90 dB@BW 0.1–20 Hz
Frequency response measurement error	< 1% (0.1 Hz–20 Hz)
Linearity measurement error	<2% (0.1 Hz–20 Hz)
Frequency Response (±3 dB)	0 Hz–80 Hz
Sampling Rates	200 Hz
Power Supply	12 V
Power Consumption	2 W
Time Service/Time Giving	GPS or NTP
Transmission Port	10/100 M Adaptive network port
Data Memory	32 G Memory card, cyclic storage
Control System	Operation system: Embedded Linux system MCU: Embedded 32-bit ARM CPU Main frequency: 400 MHz RAM: 64 MB Flash memory: 256 MB Supporting the operation of third-party software

**Table 2:** Three safety levels of a building with different structural types.

Key Parameter	Structural Type	Building Safety Level		
		Level 1	Level 2	Level 3
IDR limitation	Med-high rise steel building	IDR < 1/300	1/300 ≤ IDR < 1/50	IDR ≥ 1/50
	R.C. frame	IDR < 1/550	1/550 ≤ IDR < 1/50	IDR ≥ 1/50
	R.C. frame-shear wall, slab-column-shear wall, frame-tube	IDR < 1/800	1/800 ≤ IDR < 1/100	IDR ≥ 1/100
	R.C. shear wall, tube-tube	IDR < 1/1000	1/1000 ≤ IDR < 1/120	IDR ≥ 1/120
Structure performance	-	Elastic deformation	Elasto-plastic deformation	Severe damage
Contingency measure	-	Immediate occupancy	Occupancy after repair	Collapse prevention

## Data Availability

Generated during the study.

## Symbols and Abbreviations

$f_{d}$	=	the lower cut-off frequency
$f_{u}$	=	the upper cut-off frequency
$h_{i}$	=	the story heigh of the ith story
H(k)	=	the filter function
x(l)	=	the displacement vector
$\dot{x}(l)$	=	the velocity vector
$\ddot{x}(l)$	=	the acceleration vector
$\ddot{X}(k)$	=	the Fourier transform of $\ddot{x}(l)$
$x_{\lim}$	=	the threshold or limit of maximum displacement
$\theta_{\lim}$	=	the threshold or limit of inter-story drift ratio
$\theta_{i}$	=	the inter-story drift ratio of the *i*^th^ story
χ	=	*n*-mode tensor after missing data reconstruction
T	=	*n*-mode tensor with missing data
IDR	=	Inter-story Drift Ratio
MEMS	=	Micro-Electro Mechanical System
SHM	=	Structural Health Monitoring
